# Unruptured rudimentary horn pregnancy presenting with acute haemoperitoneum with combined intrauterine pregnancy: A case report 

**Published:** 2015-01

**Authors:** Meenakshi Lallar, Rajesh Nandal, Deepak Sharma

**Affiliations:** 1 *Department of Obstetrics and Gynecology, SHKM Medical College, Haryana, India.*; 2 *Department of Paediatrics, Artemis Health Institute, Gurgaon, Haryana, India.*; 3 *Department of Neonatology, Fernandez Hospital, Hyderabad, Andhra Pradesh, India.*

**Keywords:** *Cornual Heterotopic Pregnancy*, *Haemoperitoneum*, *Maternal mortality*, *Neonatal mortality*

## Abstract

**Background:**

The incidence of rudimentary heterotopic uterine horn pregnancy varies from 1:76,000 to 1:140,000. However the incidence of twin pregnancy i.e. intrauterine pregnancy in unicornuate uterus and its associated rudimentary horn pregnancy is estimated to be around 1 in 10 million gestations.

**Case:**

Here, we present 19 year old pramigravida women with acute haemoperitoneum with diagnosis of unruptured rudimentary horn pregnancy combined by intrauterine pregnancy. The patient was managed with emergency laparatomy and resuscitation. Rudimentary horn containing foetus was excised and intrauterine pregnancy was left untouched. The intrauterine pregnancy was supported with progesterone and tocolytics and the patient delivered a newborn of 2.8 kg through spontaneous labor at 37 weeks of gestation.

**Conclusion:**

Heterotopic pregnancies incidence have increased in comparison to past and there should be high level of suspicion for this rare event as this is often associated with high maternal and fetal morbidity and mortality where diagnosis is difficult and challenging and easily missed. . Timely intervention provides survival of intrauterine pregnancy in case of twin pregnancy, even in low resource settings where usually the diagnosis is missed before acute event.

## Introduction

The incidence of rudimentary uterine horn pregnancy reported in literature varies from 1:76,000 to 1:140,000 with around 600 cases reported worldwide till yet (1, 2). However the incidence of heterotopic twin pregnancy i.e. intrauterine pregnancy in unicornuate uterus and its associated rudimentary horn pregnancy is estimated to be around 1 in 10 million gestations. Only five cases of such pregnancies have been reported till yet in literature with two cases reporting one surviving neonate (in the unicornuate horn) and only one case report in which both the twins survived (unicornuate uterus and rudimentary horn) (3-7). Here we discuss a case of intrauterine pregnancy with co-existent rudimentary horn pregnancy, which is the sixth such case in literature and the third case reporting survival of neonate.

This is the first case reported from developing world countries where the fetus survived despite the condition being undiagnosed. It is presenting with acute haemoperitoneum at 15 weeks,that was not due to rupture of rudimentary horn, which is usually the case, but due to rupture of a vessel running superficially over the rudimentary horn.

## Case report

A 19-year-old unbooked primigravida at 15 week period of amenorrhea presented with lower abdominal pain and giddiness. The present pregnancy was a result of spontaneous conception with married life of 8 months. She had a history of slight bleeding per vaginum and lower abdomen pain at 12 weeks when a transabdominal ultrasound was done by a technician and reported normal, for which the report was not available now. On examination, she had a pulse rate of 116 beats per minute and blood pressure of 90/60 mmHg of right upper limb. The abdomen was tender and uterus was deviated to left side corresponding to the size of a 20 week pregnancy. The cervix was firm, 2.5 cm long, posterior and the cervical os was closed. A speculum examination did not reveal any cervical or vaginal pathology. No vaginal bleeding was noted. 

A transabdominal ultrasound scan was done and revealed two live fetuses, one live fetus with placenta was surrounded by momentum and the other live fetus with no surrounding myometrium and placenta was seen. Gestational age corresponded to about 16 weeks and free fluid was seen in the pelvis and Morrison’s pouch. Her haemoglobin was 9.0 g/dl, platelet count was 220x10^9^ L, and the clotting profile was normal. Ruptured tubal (cornual) ectopic pregnancy with an intrauterine pregnancy, twin pregnancy in bicornuate uterus and twin pregnancy occupying the unicornuate uterus and its associated rudimentary horn were considered as differential diagnosis. 

**Figure 1:**
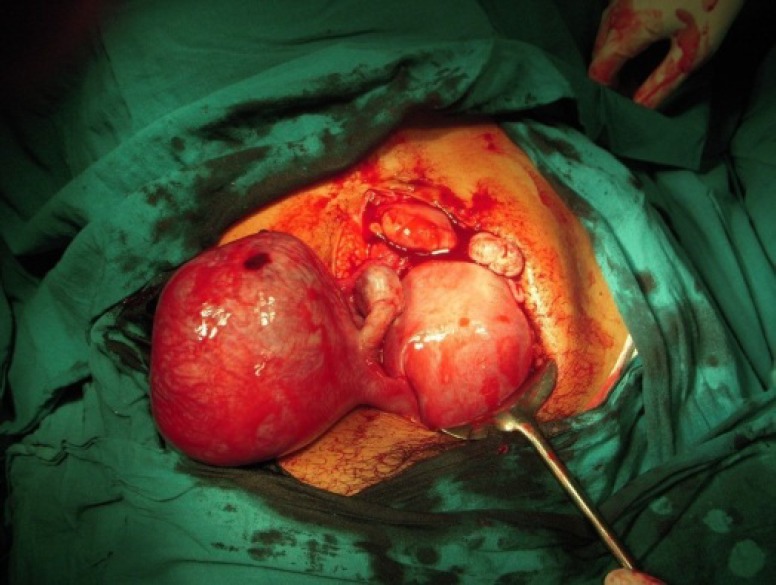
Unicornuate uterus of 16 weeks gestation with intact left rudimentary horn pregnancy of around 16 weeks. A vessel running superficially over the rudimentary horn had been torn probably due to expansion of the horn leading to haemoperitoneum

An emergency laparotomy was performed immediately with simultaneous ongoing resuscitation. Intraoperative findings revealed a unicornuate uterus of 16 weeks gestation with intact left rudimentary horn pregnancy of around 16 weeks. A vessel running superficially over the rudimentary horn had been torn, probably due to expansion of the horn (Figure 1). Around 1L of haemoperitoneum was drained. Non communicating rudimentary horn was attached to the main uterus with a fibro muscular band which was then excised. Both the ovaries and the Fallopian tubes were normal. The intrauterine pregnancy was left untouched. 

**Figure 2:**
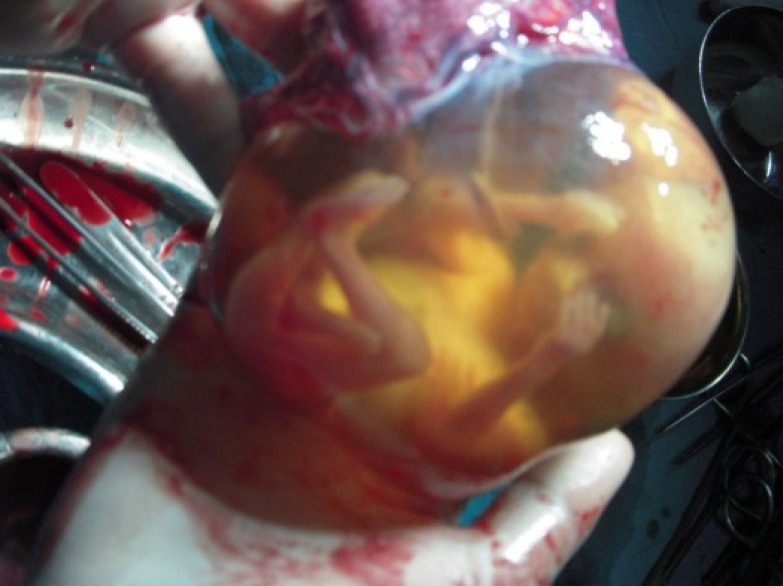
Intact foetus inside the rudimentary horn which was excised

Three units of packed cell blood transfusion were given intraoperatively. The intrauterine pregnancy was supported with progesterone and uterine relaxants given intraoperatively and progesterone was continued up to 4 weeks postoperatively. The postoperative period was uneventful. The patient went into spontaneous labor at 37 weeks of gestation and delivered a healthy baby boy weighing 2.8 kg. There were no postnatal maternal and neonatal complications. On histopathological examination sectioning showed a fetus with a crown-rump length of 122 mm. Microscopy Sections showed normal villi within a thickened smooth muscle cavity. Few endometrial glands and stroma were seen within the muscle layer. The findings were consistent with rudimentary horn pregnancy (Figure 2). Consent from the patient was taken for publication of case report.

**Differential diagnosis**

• Ruptured tubal (cornual) ectopic pregnancy with an intrauterine pregnancy

• Twin pregnancy in bicornuate uterus 

• Twin pregnancy occupying the unicornuate uterus and its associated rudimentary horn. 

## Discussion

The reported incidence of Mullerian duct anomalies is estimated to be around 4.3% and of unicornuate uterus is 0.4% (1 in 20,000 to 60,000 fertile women) (8). Unicornuate uterus with a rudimentary horn is a congenital uterine anomaly which result from developmental arrest of the two Mullerian ducts with simultaneous incomplete fusion of the opposite side. According to American Fertility Sciety Unicornuate uterus is classified in class II Mullerian anomaly i.e. a disorder of lateral fusion of Mullerian ducts and further sub grouped to communicating and non- communicating rudimentary horn (90%) types (9). The connection between the uterus and the rudimentary horn can be either fibrous or fibro muscular.

Usually pregnancy in a non-communicating rudimentary horn is very rare occurrence and is thought to be product of trans-peritoneal migration of a sperm or a fertilized ovum (10). The incidence of twin pregnancy occupying both unicornuate uterus and its associated rudimentary horn is estimated to be 1 in 10 million gestations in literature and only 5 such cases have been reported in available literatures so far (3-7). A high degree of index of suspicion should be kept and should be considered as differential in pregnant females with a known uterine anomaly, or an pregnant female with an unusual pelvic ‘mass’ in pregnancy or ectopic pregnancy or abnormal uterine shape or location and abdominal pain especially in the second trimester of pregnancy. 

With the availability of newer techniques which are easily accessible like ultrasound scan, CT scan, MRI and laparoscopy diagnosis is usually made before rupture in majority of the cases. The ultrasound scan have a sensitivity of around 26%, which decreases further with advancement of gestation, hence there are chances of missing it in inexperienced hands as in the case of our patient (11). Heterotopic pregnancies incidence have increased in present world in comparison to past because of increased used of assisted reproductive techniques (12, 13). An early diagnosis of heterotopic pregnancy is very important for the intrauterine fetus and the mother. The mother who has conceived with assisted reproductive techniques should be under regular scans and follow up as most of the heterotopic pregnancies present in casualty department with haemoperitoneum and shock. Once a heterotopic pregnancy is diagnosed, the treating physician should manage it without producing danger to the intrauterine pregnancy. In an early gestation, conservative management with laparoscopy or by injecting potassium chloride solution in the ectopic gestational sac under vaginal sonography can be done and regular follow up of mother should be taken (14). 

Celiloglu *et al* in 1991 published a case report of heterotopic twin pregnancy in bicornuate uterus with a fetus in each horn. During the second trimester there was rupture of rudimentary horn which resulted in two non-viable fetus (3). Sahakian *et al* in 1992 reported case of a rudimentary horn pregnancy coexistent with an intrauterine pregnancy in the patient at 19 weeks gestation with acute abdominal pain. On laparotomy ruptured left rudimentary horn pregnancy was found which was repaired followed by onset of preterm labor after 24 hours, and second twin was delivered despite aggressive tocolysis (4). 

The first reported case in literature of fetal survival in a twin pregnancy with one twin in a rudimentary horn was described by Opinel *et al* in 1995. This heterotopic pregnancy was first diagnosed at 18 weeks and was managed conservatively on outpatient basis with close observation on an outpatient basis. In the first antenatal scan uterine wall of 5 mm was reported which got thinned out in subsequent scans. The fetus in the rudimentary horn died spontaneously at 23 weeks of gestation, and a healthy alive term neonate was born from unicornuate uterus at 38 weeks (5). 

In another case reported in 1998 by Gagnon *et al* woman presented at 17 weeks with pain abdomen. Antenatal scans showed twin pregnancy with normal weight fetus in unicornuate uterus and a growth restricted fetus in rudimentary horn with impending rupture of rudimentary horn. The woman was managed with a selective twin reduction of rudimentary horn followed by an amino-decompression of the rudimentary horn. She was followed and her pregnancy progressed uneventfully until 36 weeks of gestation when she delivered late preterm neonate by elective caesarean section (6). 

In 2003 Ejnès *et al* described first and only reported case in literature where both the siblings were successfully delivered by caesarean section(one in unicornuate uterus and other in rudimentary horn) prior to any complication (7). All above cases reported survival of the fetus but were diagnosed prior and neither of them presented with acute haemoperitoneum as in our case. As 90% of ruptures occur before 20 weeks in such twin pregnancies conservative management is little risky especially when these pregnancies are diagnosed early in gestation. Heterotopic twin pregnancies with one twin in a rudimentary horn are always dichorionic as a rule and they do not have any vascular connections between each other. 

Therefore the death or selective termination of one of twin will not influence the long term neurological and developmental outcome of the other twin like periventricular leucomalacia or cerebral palsy and it might lead to reduction in maternal and perinatal mortality and morbidity by decreasing the risk of rupture of the rudimentary horn and premature delivery (5, 6). Treatment of the ruptured horn will require an emergency laparotomy and rupture at a younger gestational age can lead to the loss of both twins as reported previously (3, 4). 

A planned surgical approach including a laparotomy with hysterotomy and possibly partial hysterectomy can also be considered as treatment option although risk associated with it includes higher incidence of bleeding and preterm labour and delivery. Our case is unique as the pregnancy continued even after patient presented with acute haemoperitoneum and underwent resection of rudimentary horn. 

## Conflict of interest

There is no conflict of interest and there was no external source of funding 
